# Hierarchical Parallelization of Gene Differential Association Analysis

**DOI:** 10.1186/1471-2105-12-374

**Published:** 2011-09-21

**Authors:** Mark Needham, Rui Hu, Sandhya Dwarkadas, Xing Qiu

**Affiliations:** 1 Department of Computer Science, University of Rochester, PO Box 270226, Rochester, New York 14627, USA; 2 Department of Biostatistics and Computational Biology, University of Rochester, 601 Elmwood Avenue Box 630, Rochester, New York 14642, USA

## Abstract

**Background:**

Microarray gene differential expression analysis is a widely used technique that deals with high dimensional data and is computationally intensive for permutation-based procedures. Microarray gene differential association analysis is even more computationally demanding and must take advantage of multicore computing technology, which is the driving force behind increasing compute power in recent years. In this paper, we present a two-layer hierarchical parallel implementation of gene differential association analysis. It takes advantage of both fine- and coarse-grain (with granularity defined by the frequency of communication) parallelism in order to effectively leverage the non-uniform nature of parallel processing available in the cutting-edge systems of today.

**Results:**

Our results show that this hierarchical strategy matches data sharing behavior to the properties of the underlying hardware, thereby reducing the memory and bandwidth needs of the application. The resulting improved efficiency reduces computation time and allows the gene differential association analysis code to scale its execution with the number of processors. The code and biological data used in this study are downloadable from http://www.urmc.rochester.edu/biostat/people/faculty/hu.cfm.

**Conclusions:**

The performance sweet spot occurs when using a number of threads per MPI process that allows the working sets of the corresponding MPI processes running on the multicore to fit within the machine cache. Hence, we suggest that practitioners follow this principle in selecting the appropriate number of MPI processes and threads within each MPI process for their cluster configurations. We believe that the principles of this hierarchical approach to parallelization can be utilized in the parallelization of other computationally demanding kernels.

## Background

Microarray gene differential expression analysis has been widely used to uncover the underlying biological mechanism. Researchers utilize this technology to identify potentially "interesting" genes. More specifically, a statistical test is applied to each individual gene to detect whether the mean expression level of this gene is the same or not across different biological conditions or phenotypes studied in an experiment. A chosen multiple testing procedure (MTP) is then employed to control certain per-family Type I errors. Genes work together to fulfill certain biological functions and they are known to be strongly correlated [1,2]. The structure of inter-gene correlation contains rich information that cannot be extracted from mean expression levels. Recent years have seen more and more research focusing on gene dependence structures. For example, some procedures, such as gene set enrichment analysis [3,4], incorporate existing biological gene sets information into statistical procedures. Gene cluster analysis uses gene dependence and similarity to group genes [5-11]. Gene network analysis, such as method based on Gaussian or Bayesian networks, employs gene dependence to study gene dynamics and reasoning [12-14]. Another approach is to directly select genes based on the phenotypic differences of their dependence structure [15-20]. In this paper, we consider the very last approach and focus on a gene differential association analysis (henceforth denoted as GDAA) procedure proposed in [19]. Unlike traditional differential gene expression analysis, GDAA is designed to select genes that have different dependence structures with other genes in two phenotypes. It complements the analysis of differentially expressed genes. Combining both gene differential association analysis and gene differential expression analysis provides a more comprehensive functional interpretation of the experimental results. As an example, GDAA was applied in [20] to two sets of Childhood Leukemia data (HYPERDIP and TEL) [21] and selected differentially associated (DA) genes that could not be detected by differential gene expression analysis. Furthermore, the TEL group is differentiated from other leukemia subtypes by the presence of t(12;21)(p13;q22) translocation, generating the TEL-AML1 fusion gene. Through the over-representation of DA genes, the chromosomal band 21q22.3 containing the TEL-AML1 fusion gene was identified. This chromosomal band was not identified by differential gene expression analysis.

A typical microarray data set reports expression levels for tens of thousands of genes. For example, both sets of Childhood Leukemia data HYPERDIP and TEL [21] have expression levels for *m *= 7, 084 genes updated from the original expression levels by using a custom CDF file to produce values of gene expressions. The CDF files can be found at http://brainarray.mbni.med.umich.edu. Please see [19] for more details. Each slide is then represented by an array reporting the logarithm (base 2) of expression level on the set of 7,084 genes. For convenience, the words "gene" and "gene expression" are used interchangeably to refer to these gene expressions in this paper. Due to such a high dimensionality, the computation of traditional gene differential expression analysis is considered to be more time consuming than many traditional statistical analyses in medical research. A gene selection procedure based on gene dependence structures has to be even more computationally intensive. This is because the dependence structure is typically measured by a pertinent association score, such as the Pearson correlation coefficient for all gene *pairs*, of which the multiplicity (dimensionality) is $\frac{m\left(m-1\right)}{2}$ instead of *m*. It is therefore more computationally intensive to detect the differences hidden in the correlation matrix. In particular, for the procedure proposed in [19], the length of the computation is *O*(*m *× *m *× *n *× *K*), where *m *= 7, 084 is the number of genes, *n *= 79 is the number of subjects in each phenotypic group, and *K *= 10, 000 is the number of permutations for approximating the statistical null distribution. Such large number of permutations is necessary because statistical inference for microarray analysis is based on multiple testing adjusted *p*-values, which demands much finer estimation of unadjusted *p*-values compared to regular permutation tests. With a large number of genes and a medium sample size, running GDAA can take several days or even a month. For example, a sequential implementation of the procedure in [19] took nearly two months to complete the calculation on a computer with a 2 GHz AMD Opteron processor and 2GB SDRAM. Until about 2003, processor designers were able to leverage technology advances that allowed increasing numbers of smaller and faster transistors on a single chip in order to improve the performance of sequential computation. Hence, it was possible for computational scientists who wanted their codes to run faster to simply wait for the next generation of machines. However, the reality is that around 2003, chipmakers discovered that they were no longer able to sustain faster sequential execution due to the inability to dissipate the heat generated by the computation [22]. Consequently, designers turned to using the increasing transistor counts to add more processors, each of which execute independent sequential computation. The processors typically share access to the memory subsystem and off-chip bandwidth. These *multicore *chips now dominate the desktop market and are used to put together multiprocessor servers consisting of multiple processor chips, as well as networked clusters of such servers for high-end computation. Parallel computing (utilizing multiple compute resources simultaneously for the same application) that effectively leverages these increasingly multicore clusters of multiprocessors is thus even more critical than in the past in order to obtain results in a timely manner.

In this paper, we propose a new parallel design for the gene differential association analysis procedure in [19]. The key to our parallelization strategy is that it takes advantage of both fine and coarse-grain parallelism (the granularity representing the frequency of sharing/communication in the concurrent computation). The hardware-based memory sharing within a multicore is utilized for the fine-grain parallelism (with higher need for sharing/communication). Sharing memory in hardware avoids the need for data replication. Since GDAA utilizes a multivariate nonparametric test, it has more memory needs than a comparable gene differential expression analysis. Therefore, the memory sharing feature in our strategy is also critical to reducing the bandwidth demands of the GDAA procedure. The results show that our strategy leverages GDAA's characteristics to reduce the memory and bandwidth needs of the application, thereby improving computational efficiency.

## Implementation

### Gene Differential Association Analysis Procedure

We outline the related GDAA procedure below. More details can be found in [19].

#### Statistical Hypothesis Testing

Assume there are two biological conditions or phenotypes *A *and *B*. Under each condition *n *subjects are sampled, each measured with *m *gene expression levels.

We denote these gene expressions by {*x_ij_*}, 1 ≤ *i *≤ *m *and 1 ≤ *j *≤ *n*. For the *i*th gene, we first compute an (*m *- 1)-dimensional random vector **r***_i _*= (*r*_*i*1_, ⋯, *r*_*i*,*i*-1_, *r*_*i*,*i*+1_, ⋯, *r_im_*). Here *r_ik _*is the Pearson correlation coefficient between the *i*th and the *k*th gene, i.e.,

$$r_{ik}=\frac{n\sum_{l=1}^{n}x_{il}x_{kl}-\sum_{l=1}^{n}x_{il}\sum_{l=1}^{n}x_{kl}}{\sqrt{n\sum_{l=1}^{n}x_{il}^{2}-{\left(\sum_{l=1}^{n}x_{il}\right)}^{2}}\sqrt{n\sum_{l=1}^{n}x_{kl}^{2}-{\left(\sum_{l=1}^{n}x_{kl}\right)}^{2}}}.$$

Fisher transformation is then applied to these correlation coefficients:

$$w_{ik}=\frac{1}{2}\log\frac{1+r_{ik}}{1-r_{ik}},$$

where *k *= 1, ⋯, *i *- 1, *i *+ 1, ⋯, *m*. We denote the *correlation vectors *(*w*_*i*1_, ⋯, *w*_*i*,*i*-1_, *w*_*i*,*i*+1_, ⋯, *w_im_*) by **w***_i_*. This vector represents the relationship between the *i*th gene and all other genes.

For the *i*th gene, its correlation vectors under conditions *A *and *B *are denoted by **w***_i_*(*A*) and **w***_i_*(*B*), respectively. We test the null hypotheses

$$H_{i}:F_{w_{i}\left(A\right)}\left(x\right)=F_{w_{i}\left(B\right)}\left(x\right),\;\;1\leq i\leq m.$$

where $F_{w_{i}\left(A\right)}\left(x\right)$ and $F_{w_{i}\left(B\right)}\left(x\right)$ are the joint distribution functions of **w***_i_*(*A*) and **w***_i_*(*B*), respectively. If **H***_i _*is rejected, we declare the *i*th gene to be a *differentially associated *gene.

#### The N-statistic

In order to test **H***_i_*, we need to create samples of correlation vectors to mimic the joint distributions $F_{w_{i}\left(A\right)}\left(x\right)$ and $F_{w_{i}\left(B\right)}\left(x\right)$, respectively. We divide the dataset under condition *A *into$G\left(1\leq G\leq \frac{n}{2}\right)$ subgroups, each subgroup containing $\frac{n}{G}$ subjects. In order to compute correlation coefficients, every subgroup must contain at least two subjects. Sample sizes of subgroups do not have to be equal. When *G *does not divide *n*, the last few subgroups can have a slightly larger or smaller sample size. That being said, an approximately even partition of subgroups is still desirable because it leads to better statistical power than unbalanced partitions.

From these subgroups, we compute a sample of size *G *correlation vectors for the *i*th gene, denoted by **w***_i_*(*A*, *k*), 1 ≤ *k *≤ *G*. Similarly, we have a sample of size *G *correlation vectors for the *i*th gene under condition *B*, denoted by **w***_i_*(*B*, *k*), 1 ≤ *k *≤ *G*.

Next, **H***_i _*is tested by a multivariate nonparametric test based on the *N*-statistic. This statistic has been successfully used to select differentially expressed genes and gene combinations in microarray data analysis [23-26]. The *N*-statistic is defined as follows:

(1) $$\begin{array}{ll} N_{i}= & \frac{2}{G^{2}}\overset{G}{\underset{k=1}{\sum }}\overset{G}{\underset{l=1}{\sum }}L\left(w_{i}\left(A,k\right),w_{i}\left(B,l\right)\right)\;\\ & -\frac{1}{G^{2}}\overset{G}{\underset{k=1}{\sum }}\overset{G}{\underset{l=1}{\sum }}L\left(w_{i}\left(A,k\right),w_{i}\left(A,l\right)\right)\;\\ & -\frac{1}{G^{2}}\overset{G}{\underset{k=1}{\sum }}\overset{G}{\underset{l=1}{\sum }}L\left(w_{i}\left(B,k\right),w_{i}\left(B,l\right)\right),\;\\ & \; \end{array}$$

where *L *is the kernel defined by Euclidean distance, i.e.,

$$\begin{array}{c} L(w_{i}(\cdot ,k),w_{i}(\cdot ,l))=||w_{i}(\cdot ,k)-w_{i}(\cdot ,l)||\\ =\sqrt{\sum_{1\leq j\leq m,j\neq i}{\left(w_{ij}(\cdot ,k)-w_{ij}(\cdot ,l)\right)}^{2}}. \end{array}$$

The *N*-statistic can serve as a measurement of how much the inter-gene correlation structure of the *i*th gene has changed from condition *A *to condition *B*.

#### Permutation-based Null Distribution and p-value

Denote $N_{i}^{*}$ as the *N*-statistic associated with the *i*th gene. To determine the statistical significance of $N_{i}^{*}$, which is represented by a *p*-value, we need to model the null distribution of this statistic. This can be done by the following resampling method. First, we combine the gene expression data under both conditions and randomly permute subjects. Then we divide them into two groups of equal size, mimicking two biological conditions *without *differentially associated genes. By applying formula (1), we get a permutation based *N*-statistic for the *i*th gene, which can be considered as an observation from the null distribution of *N_i_*, i.e., the distribution of *N_i _*when **H***_i _*holds. Repeating this permutation process *K *times produces *K *permutation based *N*-statistics for the *i*th gene, denoted by *N_ik_*, 1 ≤ *k *≤ *K*.

*p_i_*, the permutation based *p*-value for testing **H***_i_*, is computed as the proportion of *N_ik _*that is greater than or equal to $N_{i}^{*}$:

(2) $$p_{i}=\frac{\#\left(N_{ik}\geq N_{i}^{*}\right)}{K}.$$

To control per-family error rate (PFER), we apply the extended Bonferroni adjustment [27] to the above *p*-values to obtain the adjusted *p*-values

(3) $${\tilde{p}}_{i}=p_{i}\times m.$$

The smaller ${\tilde{p}}_{i}$ is, the more likely **w***_i_*(*A*) is different from **w***_i_*(*B*), i.e., the *i*th gene changes its relationship with all other genes across conditions *A *and *B*. If ${\tilde{p}}_{i}$ is less than a pre-defined threshold, we reject **H***_i _*and declare the *i*th gene to be a *differentially associated *gene.

#### Summary of the GDAA Procedure

The above GDAA procedure can be summarized as follows:

1. Divide the subjects (slides) under each condition (*A *or *B*) into *G *subgroups such that there are approximately $\frac{n}{G}$ subjects for each subgroup.

2. For each gene, compute its correlation vectors from all subgroups. This step produces *G *correlation vectors for one gene in each condition.

3. Compute the *N*-statistic for the *i*th gene from these 2 × *G *samples using Equation(1) and record it as $N_{i}^{*}$.

4. Pool the subjects in both conditions together. Randomly shuffle the subjects, and then split them into two groups of equal size.

5. Divide the subjects in each group into *G *subgroups and compute *G *correlation vectors in each subgroup for each gene.

6. Compute the *N*-statistics for each gene based on these 2 × *G *correlation vectors.

7. Repeat steps 4 to 6 *K *times and record the permutation-based *N*-statistics as *N_ik_*, *i *= 1, ..., *m*, *k *= 1, ..., *K*.

8. Obtain the permutation-based *p*-value, *p_i_*, using Equation(2).

9. Adjust *p*-value by using Equation(3). Select differentially associated genes based on the adjusted *p*-values and a pre-specified PFER level.

## Computation

Our parallel design is implemented using Python and C++. Python is in charge of initializing data and all communication between the master process and any slave processes -- sending out computation jobs and collecting results. C++ is used to perform the actual computation within each independent process. A high-level language such as Python provides ease of use and flexibility, especially for data initialization and coordination, but at the cost of performance. By limiting the use of Python to the initialization and coordination with the slaves (where the program spends a very small percentage of its overall time) and using C++ for the computationally intensive portions of the program, we get the best of both worlds: the flexibility of Python and the performance of C++. The use of other languages such as R instead of Python is also possible. The execution proceeds as follows:

1. Read in and initialize data (performed in Python on the master process).

2. Calculate $N_{i}^{*}$, 1 ≤ *i *≤ *m*, for the unpermuted dataset using a single core (C++ code) on the master process.

3. Create *K *permutations of the original dataset; distribute the permutations *k *(1 ≤ *k *≤ *K*) to independent slave processes (performed in Python) using MPI [28]. Work is distributed at the granularity of a single permutation -- when a process completes the computation for one permutation, it requests the next permutation.

4. Each worker/slave receives a permutation (using Python), permutes its local copy of the data, and then computes the vector of *N*-statistics using C++, parallelized using the Pthreads [29] package. A total number of *P *threads are created and the per-gene computation is distributed among threads so that each thread performs the *N*-statistic computation for $\frac{m}{P}$ genes. When *m *is not divisible by *P*, each thread receives a slightly different number of genes.

5. Once an MPI process has determined that its threads have computed all *N*-statistics (*N_ik_*, 1 ≤ *i *≤ *m*) for the *k*th permutation it was assigned, it then returns them to the master MPI process.

6. The master MPI process collects all the *N_ik _*to calculate *p*-values *p_i _*(performed in Python).

Steps 1 and 2 of the algorithm are performed sequentially. To parallelize the remaining steps, we use a two-tiered approach. At the first level, we distribute the work by spawning processes from Python using MPI. One MPI process, the "master" process, is responsible for distributing different permutations to the other "slave" processes. Each slave independently permutes the gene data according to the permutation indices received from the master process and computes the *N*-statistics for the permuted data (this code is optimized C++ code). The computed values (the vector of *N*-statistics) are then returned to the master using an MPI message, where the Python code calculates the *p*-value. The key to this implementation is that the core computation is performed in optimized C++ code.

**Figure 1:**
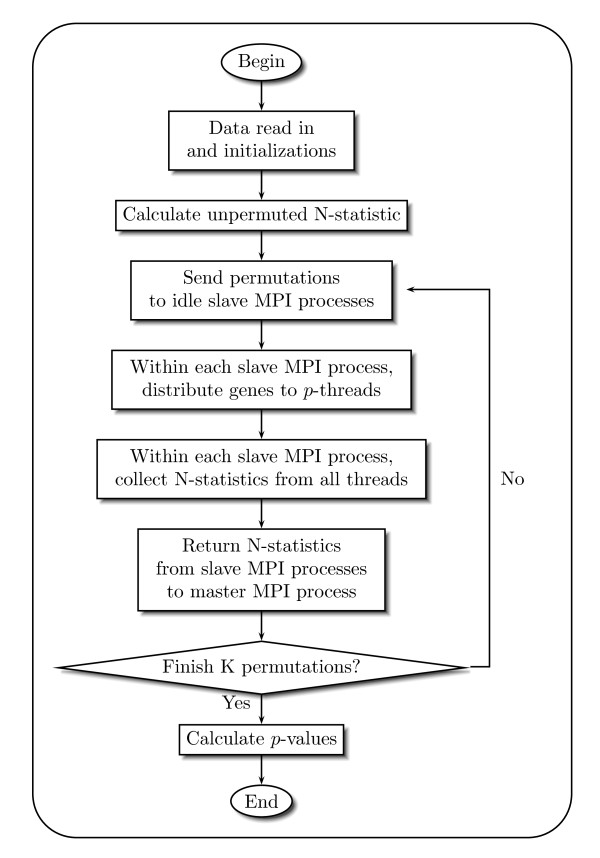
**Flowchart of hierarchical parallelization design**.

The second level of parallelization occurs within the slaves. When computing the *N*-statistics, each slave (MPI process) forks off a specified number of threads, each of which computes the permutation's *N*-statistics for a subset of genes. This allows us to vary the parallelization between MPI processes (which split the work by permutations) and threads (which divide the work by genes). For example, with one quad-core processor on a shared memory architecture, we can run one slave MPI process with four threads, 2 MPI processes each with two threads, or four MPI processes each running a single thread. Splitting the work between MPI processes versus threads has implications for performance and memory usage, which we will highlight in the evaluation section. This hierarchical design is also illustrated in the flowchart of Figure 1.

### Data Sharing

Gene expression level data in each biological condition is represented using a dynamically allocated (*m *× *n*)-dimensional array, where *n *is the number of subjects and *m *is the number of genes. This two-dimensional array is read-shared within each MPI process and its size grows as a product of *m *and *n*. There are two other dynamically allocated two-dimensional arrays created for each MPI process. These two arrays with sizes proportional to (*m *× *G*) are used for temporary storage of the correlation computation. One stores the sums of the expression levels within subgroups so that its entry in row *i *and column *j *is $\sum_{l\in \text{Subgroup}\;j}x_{il}^{\left(k\right)}$. Here $x_{il}^{\left(k\right)}$ is the expression level for gene *i *and subject *l *in the *k*th permutation. The other stores the sums of squares of the expression levels within subgroups so that its entry in row *i *and column *j *is $\sum_{l\in Subgroup\;j}{\left(x_{il}^{\left(k\right)}\right)}^{2}$. They are also read shared within the MPI process. Another two-dimensional dynamically allocated array with size proportional to *O*(*m *× *G*) is created for each thread, storing the correlation vectors for each gene. This array is both read and written by the thread. The *N*-statistic, which is a vector with the size of the number of genes *m*, is also shared across all threads within each MPI process. Each thread writes to independent regions of this vector based on the genes allocated to it.

## Results

Our evaluation is conducted on a cluster of five machines, each with 16 GBytes of memory and two 3.0 GHz quad-core Intel Xeon 5450 processors, for a total of 40 processors. The machines are interconnected using Gigabit ethernet. Each quad-core processor chip has 6 MBytes of last-level cache per pair of cores. Each machine runs Linux version 2.6.16.60-0.21-smp. The application was compiled using Python version 2.4.2, Pypar version 2.1.0, and gcc version 4.1.2 at the -O2 optimization level.

### Simulation Data

To gain better insight into the effects of different configurations on performance, we simulate several sets of data. Each set has two groups of *n *= 100 slides representing 100 subjects in each biological conditions.

Each array has *m *genes, where *m *takes on values in {1000, 2000, 3000, 4000, 5000, 6000, 7000, 8000, 9000, 10000}. The slides in each group are divided into *G *= 10 subgroups to calculate correlation vector samples. *K *= 100 permutations of the subjects in the two groups are created in order to generate the null distributions of *N*-statistics.

### Performance Analysis

**Figure 2:**
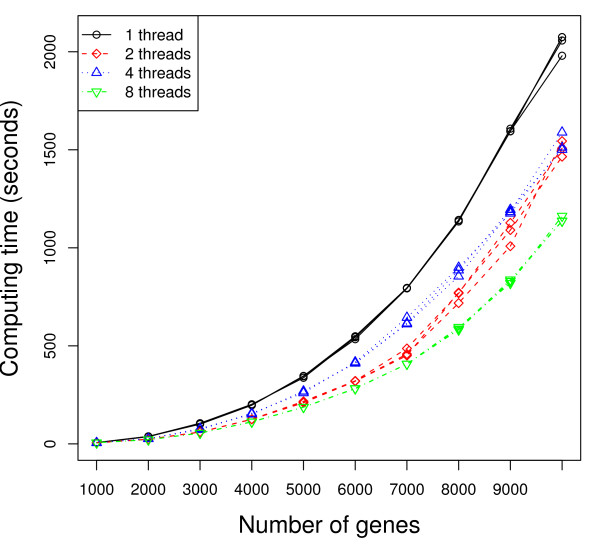
**Execution time with default OS scheduling**. Number of slides in each condition: 100. Number of permutations: 100. Numbers of MPI processes × threads: 32 × 1 (solid), 16 × 2 (dash), 8 × 4 (dot), and 4 × 8 (dash-dot).

**Figure 3:**
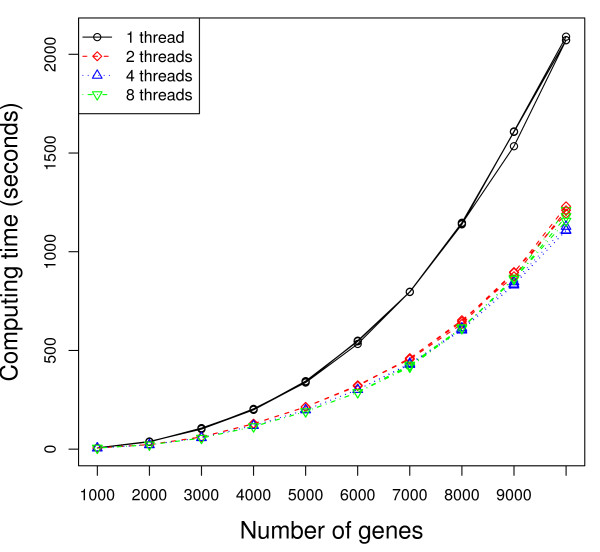
**Execution time with pinned processes**. Number of slides in each condition: 100. Number of permutations: 100. Numbers of MPI processes × threads: 32 × 1 (solid), 16 × 2 (dash), 8 × 4 (dot), and 4 × 8 (dash-dot).

**Figure 4:**
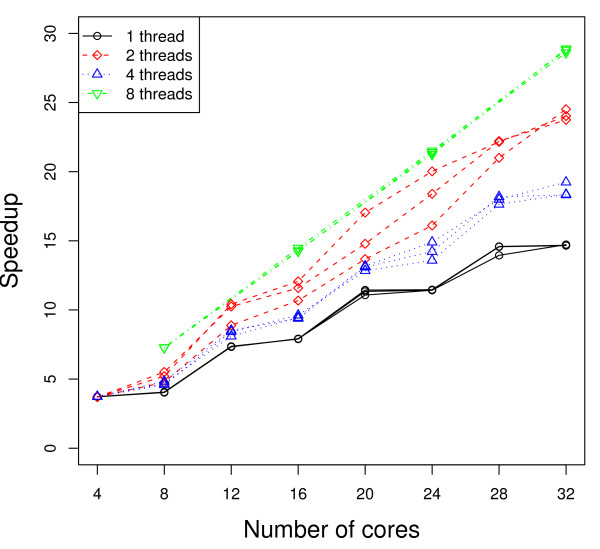
**Speedup with default OS scheduling**. Number of genes: 7000. Number of slides in each condition: 100. Number of permutations: 100. Numbers of MPI processes × threads: (number of cores) × 1 (solid), (number of cores/2) × 2 (dash), (number of cores/4) × 4 (dot), and (number of cores/8) × 8 (dash-dot).

**Figure 5:**
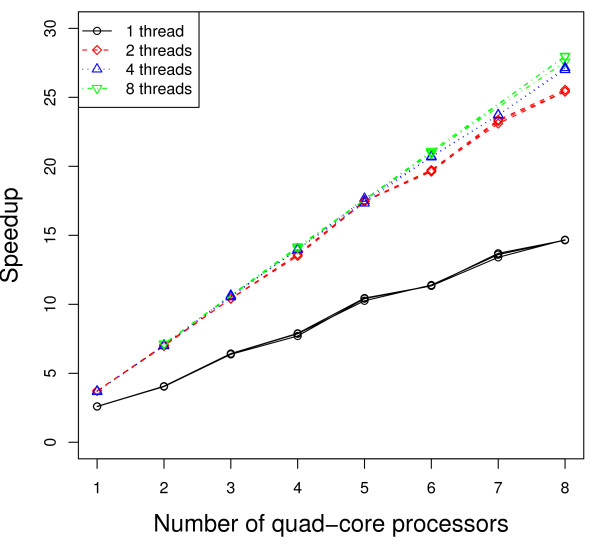
**Speedup with pinned processes**. Number of genes: 7000. Number of slides in each condition: 100. Number of permutations: 100. Numbers of MPI processes × threads: (number of cores) × 1 (solid), (number of cores/2) × 2 (dash), (number of cores/4) × 4 (dot), and (number of cores/8) × 8 (dash-dot).

Figures 2 and 3 present the execution time (measured from the time after calculation of the unpermuted statistic) as a function of the number of genes in the dataset, with the operating system default scheduling (Figure 2) and with each thread/process *pinned *(more detailed explanation to follow) so it executes only on one specific processor/core (Figure 3). While we report three times in the figures to show the variation in the results, we repeated the timing-based execution several times to ensure consistency of the results. The quad-core processor running the Python script is not used for parallel computation. The number of MPI processes forked, and correspondingly the number of threads used per MPI process, is varied. More specifically, the four sets of curves represent 32 single-threaded MPI processes, 16 dual-threaded MPI processes, 8 4-threaded MPI processes, and 4 8-threaded MPI processes, respectively. We also applied 1, 2, 4, and 8 threaded strategies to a dataset of 7000 genes while varying the number of cores (or quad-core processors) used. Figures 4 and 5 present the speedup (execution time on a single core/using a single thread divided by the execution time of the parallel implementation with the specified number of cores/threads) as the number of cores (or quad-core processors) is varied, using the operating system default scheduling (Figure 4) and with each thread/process *pinned *so it executes only on one specific processor/core (Figure 5).

**Figure 6:**
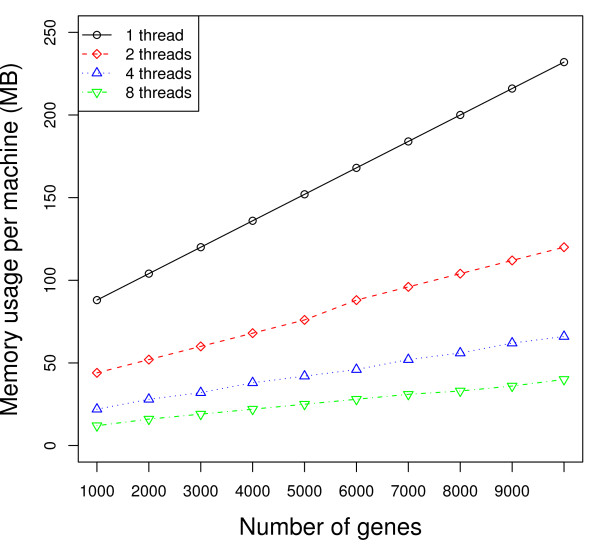
**Memory usage**. Number of slides in each condition: 100. Number of permutations: 100. Numbers of MPI processes × threads: 32 × 1 (solid), 16 × 2 (dash), 8 × 4 (dot), and 4 × 8 (dash-dot).

As shown in Figures 2 and 3, using multiple threads per MPI process outperforms the 1 thread strategy substantially. As an example, according to Figure 3, when the number of genes *m *= 10, 000, the average execution time for the 2 threaded strategy is 1211 seconds, which represents about 70% performance gain compared to the 1 threaded strategy (2077 seconds). When using only MPI processes (1 threaded strategy), there is no data sharing among the processes. All communication is strictly via messages. As the number of threads increases, Figure 6 shows that the total amount of memory required per machine goes down as the number of MPI processes decreases and the number of threads per MPI process increases. This is a result of the data sharing in the parallel threaded implementation, as described in the Data Sharing section. Parallelizing purely at the MPI level results in multiple copies of the data structures being created and exerts more pressure on the memory as well as any shared cache in the system. On our experimental platform, the last-level cache has a size of 6 MBytes, which is shared between two cores in a physical package (quad-core processor). When the working set of the processes/threads executing on these cores exceeds the capacity of the 6 MByte shared cache, some performance will be lost. Using threads allows the cores to share space in the cache more effectively and has the added benefit of reducing memory latency due to the prefetching effect of 1 core on the other. In addition, reducing the number of permutations (MPI processes) computed on at the same time reduces the pressure on the communication link with the master process, which must coordinate and communicate with each MPI process and can therefore result in a bottleneck. Any coarse-grain load imbalance at the permutation level is also mitigated. On our platform, we also observe some anomalies in behavior -- faster performance was observed using 2 threads per slave MPI process rather than with 4 threads (see Figure 2 and 4). In addition, the variance in performance across runs is high, especially in the 2 threaded runs. The 2 threaded strategy represents the sweet spot in terms of leveraging shared resources on this architecture (a 6 MByte cache shared by 2 cores), presuming that the 2 threads strategy execute on cores that share a cache. Our hypothesis is that the default operating system scheduling of the threads does not ensure this affinity. To confirm our hypothesis, we add code to force thread affinity -- each thread is *pinned *to a particular core while ensuring that threads within a process share a cache and remain within a single chip when possible. The resulting performance, shown in Figure 3 and 5, corroborates our hypothesis. The variance in performance is no longer observed. Most of the efficiency gains from sharing across threads is observed when using 2 threads, i.e., when the parallelization matches the underlying physical characteristics of the machine and leverages the shared cache between 2 cores. Additional performance benefits beyond 2 threads are small. More specifically, the 2, 4, and 8 threaded strategies show only small differences in performance once the threads are pinned to ensure cache sharing. In Figure 3, the 8 threaded strategy is a little better if the number of genes is between 3000 and 7000. Otherwise, the 4 threaded strategy shows slightly better performance. These variations across different numbers of threads come from differences in load balance at the Pthread and MPI parallelization levels.

In Figures 4 and 5, we notice that the speedup curves are not very smooth. This step function can be attributed to several causes. The first is load imbalance due to the granularity of workload distribution -- permutations at the MPI parallelization level and genes at the Pthread parallelization level. When using 1 thread per MPI process to conduct 100 permutations, as one example, with 5 processors (20 cores), each core runs 5 permutations (⌈100/20⌉). If we increase the number of processors to 6 (24 cores), some cores will still execute 5 permutations while others execute 4, so that execution time remains proportional to ⌈100/24⌉ = 5, resulting in practically no increase in speedup. As the number of permutations executed per MPI process decreases (with an increasing number of cores), the fraction of idle/wasted time on the cores with one less permutation to execute increases, resulting in lower efficiency. In the case of Figure 4, the increased scheduling variability and poor choice of scheduling when adding a quad-core processor within a machine also contributes to the step function in the 2 and 4 threaded curves. Once the scheduling is made both deterministic and ensures appropriate cache sharing, the step function is less pronounced in the multi-threaded runs in Figure 5 due to their reduced memory bandwidth demands and smoother load function at the MPI level.

## Conclusions

Microarray technology has made it possible for medical researchers to measure and study the behavior of thousands of genes at once. Technology advances have been on a fast track in recent years, making it possible to conduct microarray experiments much faster and less expensive than in the past. This trend has been leveraged with the availability of larger and larger datasets. Turning so much raw information into knowledge presents a major challenge for both statistical analysis and computation. As of now, microarray data are used for crude screening of differentially expressed genes. Exploiting the rich information contained in the inter-gene dependence structure has not become a routine, despite the availability of several gene association analysis procedures. This is largely due to the computing bottleneck.

In this paper, we present a parallelized implementation of gene differential association analysis that is designed to leverage the features of today's multicore platforms in which resources are shared among processors at a much finer granularity than in the past. We apply the conventional wisdom of parallelizing at the coarsest granularity to distribute permutations among the nodes in a cluster, using MPI for communication. In addition, we parallelize at the finer granularity of per-gene computation within a single dual quad-core machine using shared memory (Pthreads). Sharing memory across threads helps reduce demand for the shared last-level cache capacity on the chip by allowing independent threads to share a single copy of the gene expression data. Our results show that this strategy utilizes the multicore cluster platform much more effectively. In general, the performance sweet spot occurs when using a number of threads that allows the working sets of the corresponding MPI processes to fit within the machine's shared cache. We suggest that practitioners follow the principle of determining what resources are shared when making decisions on how to allocate compute resources among MPI processes and threads for their cluster machines. We believe that the principles of this hierarchical approach to parallelization can be utilized in the parallelization of other computationally demanding kernels.

## Availability and Requirements

• Project name: Hierarchical Parallelization of Gene Differential Association Analysis;

• Project home page: http://www.urmc.rochester.edu/biostat/people/faculty/hu.cfm;

• Operating system: Linux;

• Programming language: Python and C++;

• Other requirements: MPI (MPICH2 or Open MPI), Python, C++ Compilation tools, SWIG, Numpy, Pypar;

• Licence: GNU GENERAL PUBLIC LICENSE, Version 2, June 1991;

• No restrictions to use by non-academics.

## Abbreviations

GDAA: Gene Differential Association Analysis; MTP: Multiple Testing Procedure; MPI: Message Passing Interface; Pthreads: POSIX Threads.

## Authors' contributions

The basic idea was first proposed by RH, SD, and XQ. The detailed study design was developed by all members of the research team. MN carried out the needed computations and simulations and the majority of the software development. All authors have read and approved the final manuscript.
